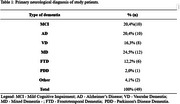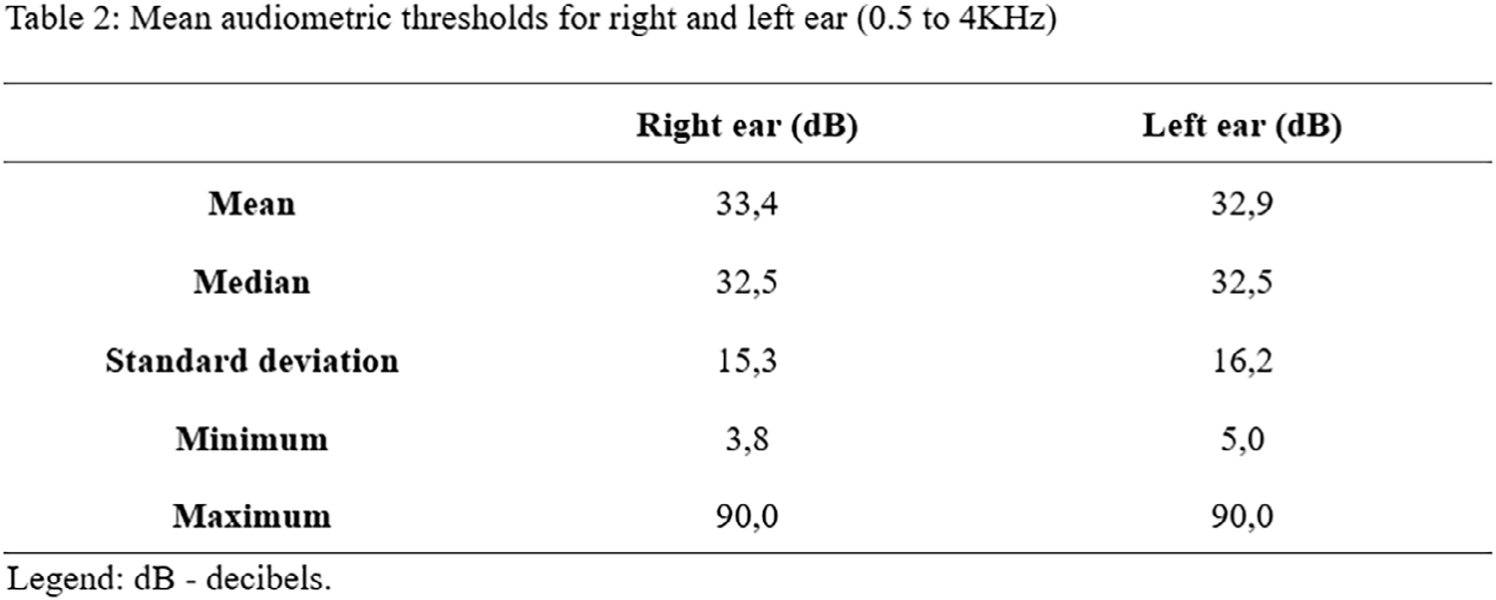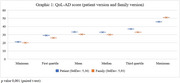# Relationship between hearing acuity and self‐perception of quality of life in patients with cognitive decline

**DOI:** 10.1002/alz70858_105977

**Published:** 2025-12-26

**Authors:** Mariane Gomes Machado, Thais Helena Machado, Laís Ferreira Alves, Paulo Caramelli, Luciana Macedo de Resende

**Affiliations:** ^1^ Universidade Federal de Minas Gerais, Belo Horizonte, Minas Gerais, Brazil; ^2^ Faculty of Medicine ‐ Federal University of Minas Gerais, Belo Horizonte, Minas Gerais, Brazil

## Abstract

**Background:**

Quality of life (QoL) can be influenced by cognitive status, sociodemographic factors, and other health issues, such as hearing loss. The aim of this study was to analyze QoL, using the Quality of Life in Alzheimer's Disease (QOL‐AD) questionnaire, in relation to cognitive status, hearing acuity, other comorbidities, and sociodemographic data.

**Method:**

Cross‐sectional study, with convenience sampling, conducted in a Cognitive and Behavioral Neurology outpatient clinic with cognitive decline patients who could understand and execute commands, who did not use hearing aids, and who had a caregiver available. Participants underwent tonal audiometry and the QOL‐AD questionnaire in interview format for patients and caregivers, with additional clinical data collected from medical records.

**Result:**

Final sample consisted of 32 men (65.3%) and 17 women (34.7%), ages ranging from 30 to 89 years. In addition to the cognitive health impairment (Table 1), most patients had general clinical health comorbidities (79.6%), and 24.5% had psychiatric comorbidities. The mean MMSE was 19.75 (±5.92), indicating variability in cognitive decline. The mean education level was 6.61 years. The QoLAD composite score had a mean of 32.19 points (±4.76), and ranged from 22.33 to 43.33 points. Normal hearing was detected in 24.5% of patients, while 40.8% had mild hearing loss, 26.5% had moderate hearing loss, 6.1% had moderately severe hearing loss, and 2.0% had profound hearing loss. Mean audiometric thresholds are shown in Table 2. The analysis revealed that the presence of health comorbidities significantly impacts the QoL of patients, especially when psychiatric conditions are involved. Functionality and mental health are critical aspects for QoL, outweighing the influence of cognitive status alone. No significant relationship was found between hearing acuity and QoL. Although, with 75.4% of patients presenting some degree of hearing loss and not using hearing aids, there is an opportunity to provide hearing care for these patients, considering the socio‐emotional impact that hearing loss can generate.

**Conclusion:**

Future clinical trials should investigate the extent to which auditory interventions contribute to improving the QoL of individuals with cognitive decline.